# Reliable Numerical Models of Nickel-Titanium Stents: How to Deduce the Specific Material Properties from Testing Real Devices

**DOI:** 10.1007/s10439-022-02932-1

**Published:** 2022-02-25

**Authors:** Francesca Berti, Sara Bridio, Giulia Luraghi, Sanjay Pant, Dario Allegretti, Giancarlo Pennati, Lorenza Petrini

**Affiliations:** 1 Laboratory of Biological Structure Mechanics (LaBS), Department of Chemistry, Materials and Chemical Engineering “Giulio Natta”, Politecnico di Milano, Piazza Leonardo da Vinci 32, 20133 Milan, Italy; 2 Zienkiewicz Centre for Computational Engineering, Engineering Central, College of Engineering, Swansea University, Bay Campus, Swansea, SA1 8EN UK; 3 Department of Civil and Environmental Engineering, Politecnico di Milano, Piazza Leonardo da Vinci 32, 20133 Milan, Italy

**Keywords:** Digital twin, Surrogate modeling, Material identification, Self-expandable stent, Model validation

## Abstract

The current interest of those dealing with medical research is the preparation of digital twins. In this frame, the first step to accomplish is the preparation of reliable numerical models. This is a challenging task since it is not common to know the exact device geometry and material properties unless in studies performed in collaboration with the manufacturer. The particular case of modeling Ni–Ti stents can be highlighted as a worst-case scenario due to both the complex geometrical features and non-linear material response. Indeed, if the limitations in the description of the geometry can be overcome, many difficulties still exist in the assessment of the material, which can vary according to the manufacturing process and requires many parameters for its description. The purpose of this work is to propose a coupled experimental and computational workflow to identify the set of material properties in the case of commercially-resembling Ni–Ti stents. This has been achieved from non-destructive tensile tests on the devices compared with results from Finite Element Analysis (FEA). A surrogate modeling approach is proposed for the identification of the material parameters, based on a minimization problem on the database of responses of Ni–Ti materials obtained with FEA with a series of different parameters. The reliability of the final result was validated through the comparison with the output of additional experiments.

## Introduction

**Figure 1:**
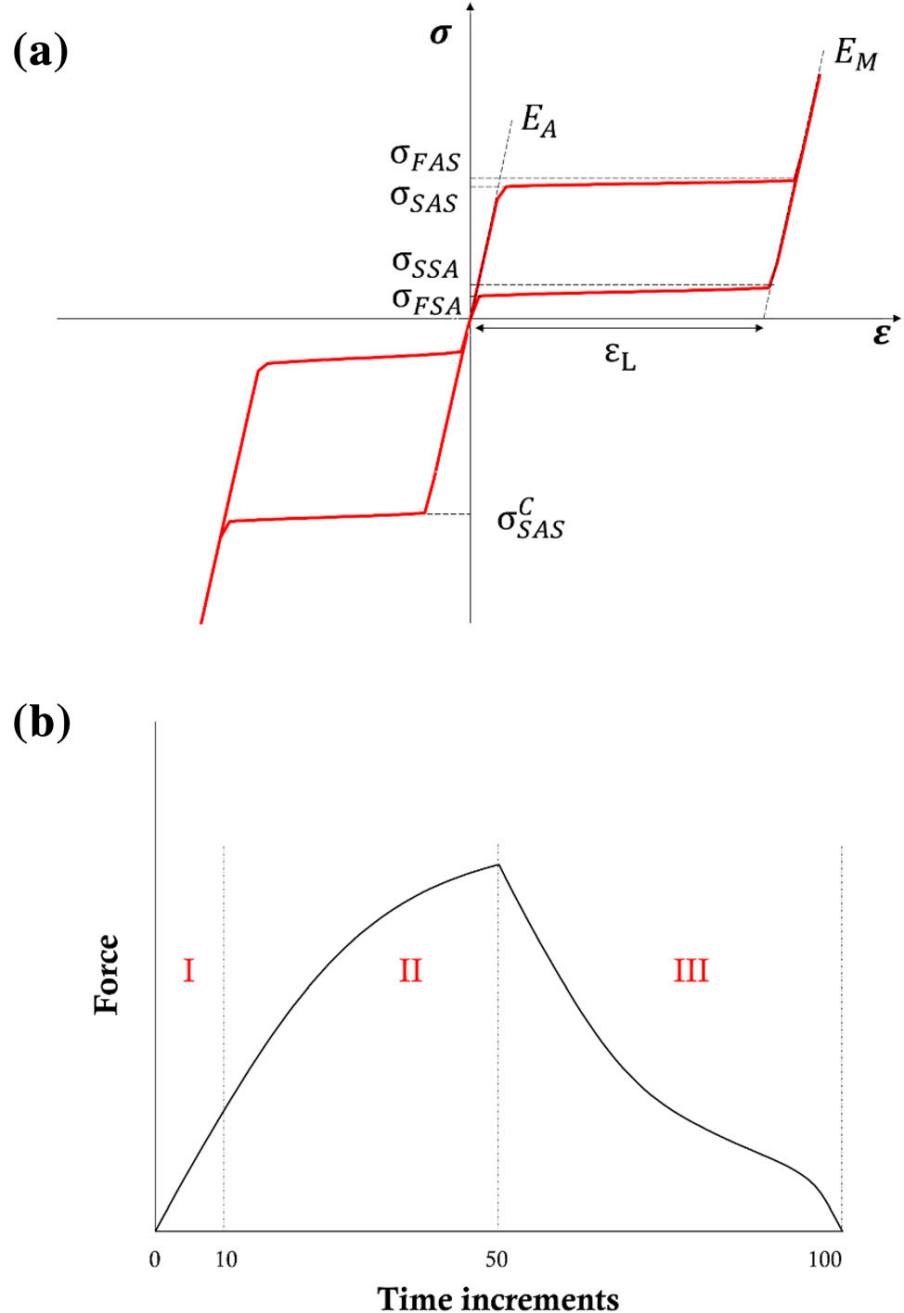
(**a**) Ni–Ti stress/strain curve in tension and compression according to the Abaqus super-elastic material module. See Nomenclature for the meaning of each parameter; (**b**) reference behavior for the virtual case: the global force was plotted against the number of time increments. Phase I is defined as the first 10 increments, Phase II is comprised between the 11th and the 50th increment while Phase III lasts from the 51st to the end (100th).

Nickel–Titanium (Ni–Ti) alloys are widely employed for the manufacturing of self-expandable devices, such as peripheral stents. This is due to a super-elastic behavior at body temperature, allowing them to withstand considerable deformations (up to 10%) due to gait,^20^ which is related to the change between two solid phases in the lattice, namely the austenite, which is present at low strains, and the single-variant martensite present at high strains.^25^ When stress is applied to Ni–Ti, and after a modest elastic deformation of the austenite, the material reacts to the applied stress by changing its crystal structure. This “stress-induced” phase transformation allows the lattice to re-orient itself as a direct response to the load, then reverting to the original structure as the stress is removed. Looking at the loading and unloading curves of a Ni–Ti sample in tension/compression it is possible to recognize plateaus, along which large deformations can be accumulated on loading, or recovered on unloading, without significant increase, or decrease, respectively, in stress (Fig. 1a).^36^

Peripheral Ni–Ti stents are the gold standard for the treatment of atherosclerotic disease and are characterized by complex and brand-specific designs that aim at addressing different requirements in terms of radial stiffness and flexibility,^5,21^ trying to improve the clinical outcomes that are affected by the incidence of fatigue failure.

Recently, simulations have been accepted by the regulatory authorities to support the evaluation of performance and reliability before the marketing authorization of a new medical device. Indeed, simulations are a very useful tool for improving design/development phases, integrating data from the bench, *in vivo*, and clinical studies.^38^ In this way, it is possible to reduce costs associated with several prototypes manufacturing and experimental testing. To this aim, the real object is paired with its digital version, which is designed to mimic the actual functional behavior in a virtual but realistic scenario.

Currently, there is an important scientific interest in the definition of good practices for the preparation of reliable, accurate, and credible models.^4,23,37^ Among others, two key aspects have to be carefully considered, namely the device's geometrical features and the material properties. In the specific case of stents, the geometry reconstruction through CT images^9^ or optical observation^12,28^ is quite an established and reliable method for CAD preparation, also simplified by the presence of repetitive units in the stent cells.

On the other hand, the knowledge of the mechanical properties of the constituent material remains a more challenging task, especially in the case of Ni–Ti alloys that exhibit complex non-linear behavior. Indeed, there are many examples in the literature of user subroutines that have been developed ad hoc to describe the Ni–Ti phenomenology in a very realistic way but requiring a considerable number of input parameters, and, consequently, experiments for their assessment.^18,27^ Commercial FE software such as Abaqus (Dassault Systèmes) and Ansys Mechanical (ANSYS) offer a good compromise, with a super-elastic material module in which seven parameters faithfully represent the material tensile behavior, while the typical tension-compression asymmetry is described in a very simplified manner by one parameter only (Fig. 1a).^6^

In the literature, there are many examples of Ni–Ti stents models used for investigating their static and fatigue performances.^7,8,11,15,29^ The most common approach to prepare such simulations is to take the Ni-Ti material parameters from the literature.^3,11,22,26,29^ However, even if the chemical composition of the alloy used for medical application is quite similar, these sets of parameters exhibit a great variability, which affects the credibility of the results. Indeed, each manufacturer develops ad hoc heat treatments to perform on the source tubes for guaranteeing proper transformation temperatures and the desired mechanical properties.^13^

As an alternative to literature data, multi-wires specimens can be laser-cut from the same tube of the stents and then shared the same heat treatments, as in a previous work of the authors.^1^ The wire dimensions are chosen to be in the same order as the stent v-struts to avoid any scaling effect. Given that is not trivial to have access to such samples (unless in collaboration with the manufacturer, as in the case of ^1^, or being the manufacturer itself), these specimens allow the characterization of the material properties through uniaxial tensile tests. However, it is known that Ni–Ti alloys exhibit a tension/compression asymmetry that is intrinsically related to the structure of the lattice,^10,31^ and slender specimens such as multi-wires do not allow the characterization in compression, suffering structural instability. On the other hand, the knowledge of the compressive behavior is rather crucial when dealing with stents, whose deformation fashion is mainly based on the bending of the v-struts. At this stage, unless in the case of a collaboration with the manufacturer that can provide ad hoc samples for mechanical testing, those who wish to develop an accurate model of a commercial device can base their investigation exclusively on what can be obtained by studying the device itself.

This study aims to propose a strategy that allows the preparation of credible Ni–Ti stent models, based on the identification of Ni–Ti material parameters from non-destructive experimental tests on the whole device using Finite Element Analyses (FEA) through commercial software Abaqus.

Two different stent designs, resembling commercially available ones, are used in the process. Simple experiments, such as uniaxial tensile and crush tests, are used for the development and verification of the method, respectively.

A surrogate modeling approach is proposed for the identification of the material parameters, based on a database of responses of Ni–Ti materials obtained with FEA with a series of different parameters. The unknown material parameters are identified by solving a minimization problem on the surrogate response surface.

First, two sets of tensile test simulations in which the material parameters were taken from the literature has been run; the identification process was then developed and tested on these *virtual* and known cases. Then, the method has been applied for the identification of material parameters from two real cases, involving the tensile testing of two stent designs resembling commercially available ones. Finally, a crush test has been performed on the same stent designs and the previously identified sets of parameters have been used to compare the numerical outputs with the experimental data: in this way, it was possible to provide proof of the credibility of the results under different loading conditions. The limitations of the method related to the use of the commercially-available material module for describing super-elasticity have been highlighted.

## Materials and Methods

### Workflow of the Study

In this study, a displacement-controlled uniaxial tensile test was chosen as a non-destructive simple but effective test for obtaining the macroscopic stent behavior that contains all the information on the material parameters. To do so, it was important to verify that the force-displacement curve, after an initial linear portion, showed a flattening and then, at unloading, hysteresis: this is typical of Ni–Ti devices, meaning that a consistent number of elements have entered the transformation plateau during the loading phase. While this condition is depends on the stent design, which differently affect local deformations, it usually happens when the displacement applied at the stent extremities is in the order of half the stent length. In these conditions, due to the super-elasticity of Ni–Ti stents, this test could be performed without permanently damaging the stents. On the other hand, an excessive tension could cause some elements exiting the transformation plateau, first, and then permanent yield, which results in hardening in the force-displacement plot. Starting from the observation of the Ni–Ti stress-strain curve, it was possible to recognize how the different phases of the material response could be described by a progressively increased number of parameters: as an example, in the initial frames of a tensile test, the material response is fully described by E_A_, needing the addition of few parameters only when the deformation increases and the material starts the transformation (e.g. to describe the loading plateau in the stress-strain curve). Until the removal of the applied load, the parameters describing the unloading plateau are not recruited.

Exploiting this particular feature of Ni–Ti, the identification process^14,17^ was decomposed into three phases to separately estimate the parameters affecting each phase (see Nomenclature) (Fig. 1b). Having in mind that in each simulation the output variables were saved in 100 equally spaced increments, the three phases were defined as follow:

- Phase I: the elastic response of the austenite, described by Young’s modulus E_A_. It was assessed as the first 10 time increments of the simulation (meaning up to 20% of the maximum applied displacement). For parameter sampling to construct the surrogates, all the remaining parameters were set randomly as they do not influence this phase;
- Phase II: the transformation phase during loading, described by three parameters: *σ*_SAS_, *H*, and *α*. It was assessed as the interval between the 11th and 50th time increment of the simulation (from the end of the elastic response up to the maximum applied displacement). For parameter sampling, *ε*_L_ was set equal to 0.1 to force all the elements to remain in the loading plateau, and *E*_M_ was set equal to *E*_A_;
- Phase III: the unloading phase, described by four parameters: *E*_M_, *σ*_SSA_, *σ*_FSA_, and *ε*_L_. It was assessed as the interval between the 51st and the 100th (last) time increment of the simulation.

The numerical simulations involved in the identification process were performed in the Abaqus 2019/Standard environment (Dassault Systemes, SIMULIA Corp., RI). The numerical models mimicked the experiments in terms of stent design and applied boundary conditions, discussed in “Stents FE models and functional units” section. The implemented super-elastic material module calls for eight material parameters, discussed in detail in “Ni-Ti constitutive parameters” section.

First, the identification process has been applied to two virtual cases, considering as target curves the outcome of two numerical simulations, where the set of constituent parameters was known. This allowed the development of the method in a known case as described in “Identification process on virtual cases” section.

After this, the method was used for identifying the parameters from the uniaxial tensile test on two stent designs, resembling commercially available ones. The experimental value of the force was saved during the test and used as the target for the identification as described in “Uniaxial tensile tests on real devices” section.

Finally in “Crush tests and verification” section, to prove the reliability of the identified set in describing the stent behavior even under different loading conditions, a different experiment was performed on the stents, namely a crush test. A numerical simulation mimicking this experiment was performed on both the stent designs and the force-displacement output was compared with the corresponding experimental data.

### Stents FE Models and Functional Units

**Figure 2:**
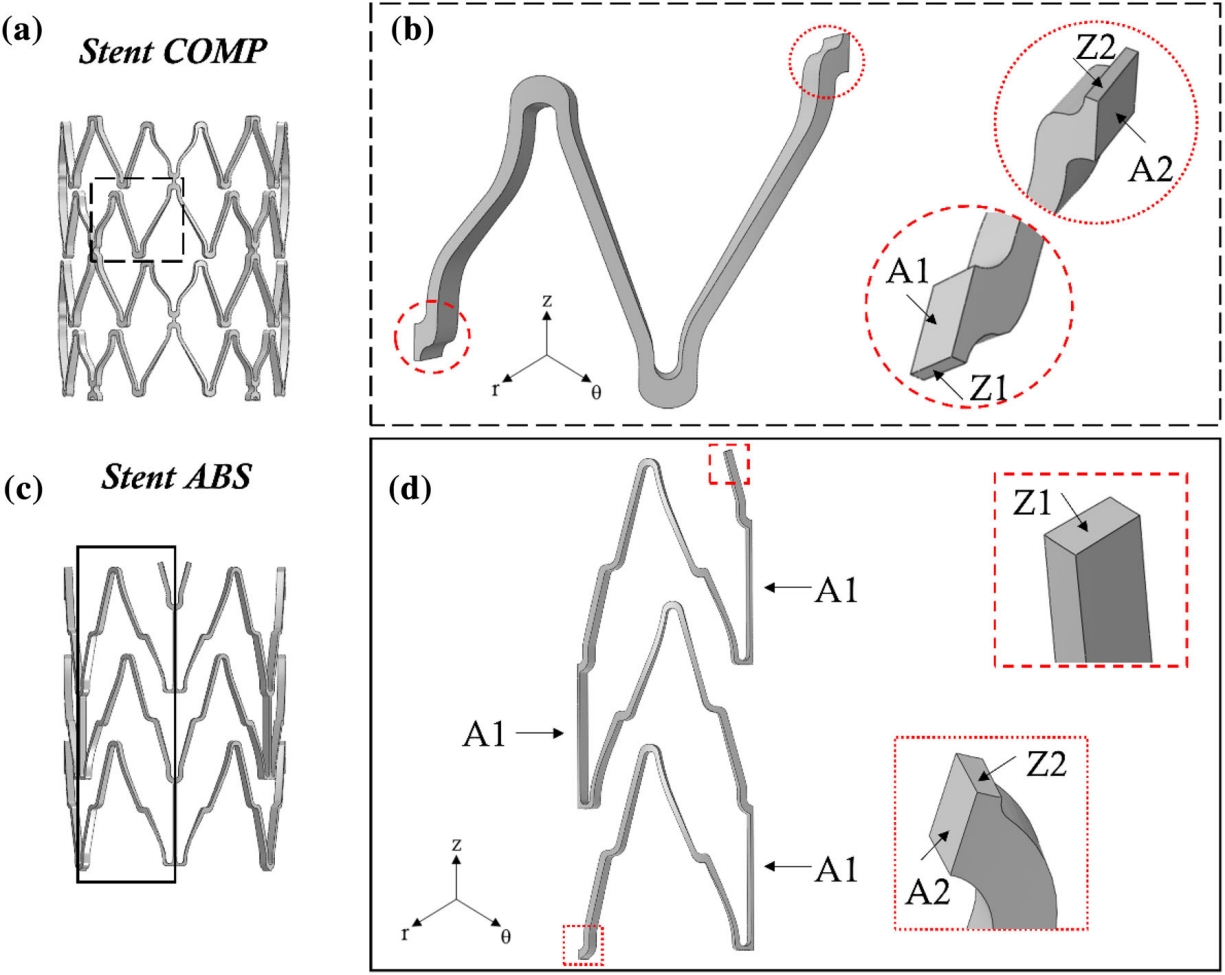
(**a**) The stent COMP geometry with detail of (**b**) the functional unit; (**c**) the stent ABS and (**d**) its functional unit. For both stents, insight into the constrained surfaces of the functional units is given.

The first stent here considered resembles the Complete® SE (Medtronic Vascular, Santa Rosa, CA, in the following COMP) and it is composed of four rings of 8 mm length, connected peak-to-peak by small links; the rings are composed of struts about 2 mm in length, 200 *µ*m thick and 100 *µ*m wide, in a v-shaped assembly (Fig. 2a). The second stent design resembles the stent Absolute Pro® (Abbott Vascular, Santa Clara, CA, in the following ABS) and it is composed of three rings connected peak-to-valley by straight links: the total longitudinal length is about 9 mm and the struts are of the same dimensions as the previous stent (Fig. 2c). For both the stents the inner diameter in the expanded configuration is 6 mm. The CAD designs of both ABS and COMP stents were reconstructed and then discretized using 196890 and 203320 8-nodes fully integrated solid elements with incompatible mode formulation, respectively, according to the sensitivity analysis performed in Ref. 1 Different mesh refinements were compared in terms of first principal stress and strain at the maximal loaded points of the structures and global reaction force when the model was axially tensioned. The optimal mesh density (considering a 5 × 5 refinement in the cross-sections of each strut) was a compromise between computational cost and result accuracy.

**Figure 3:**
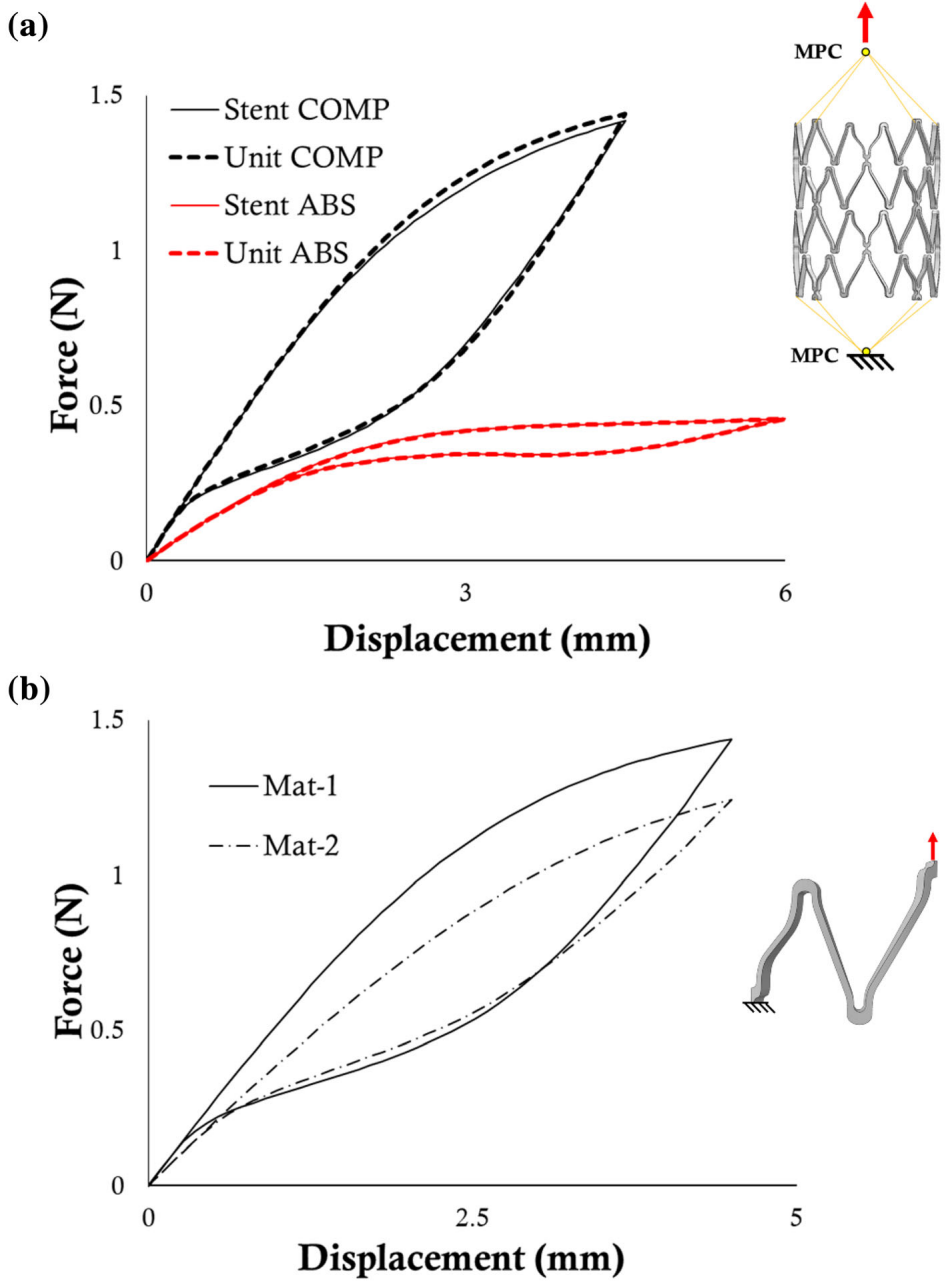
(**a**) Tensile force-displacement curves of stent COMP (black) and ABS (red), with a comparison between the numerical output obtained by simulating the whole stent (solid line) or the functional unit (dashed line) properly scaled; (**b**) tensile force-displacement curves obtained through the simulation of the COMP functional unit associated with Mat-1 and Mat-2.

Experimental data of uniaxial tensile tests on the designs were taken from a previous study.^1^ Following the same boundary conditions of the experimental tests, the stent COMP was tensioned up to 4.5 mm, while the stent ABS up to 6 mm: this is motivated by the different overall stiffnesses and by the need of obtaining a hysteresis in the curve, as already motivated in the previous section (Fig. 3a, stent COMP in black and ABS in red).

However, to fulfill the purpose of this work, a considerable number of simulations was required, which posed the attention to the need of reducing the computational time of the test (COMP: about 2 hours, ABS: about 14 hours, referring to a complete cycle of loading and unloading on a computer node with 8 CPUs Xeon CentOS 6.5 with 23 GB of RAM).

As in most stent-like devices, a minimum geometrical unit can be recognized, whose repetition along the circumferential and longitudinal directions gives the whole stent pattern. This unit was exploited during the model preparation, regarding the CAD design and the discretization phases. The stent geometrical unit not always coincides with the functional unit, defined as the minimum portion that, if properly constrained, is representative of the stent behavior under specific testing conditions. Hence, different functional units can be deducted from the same stent design according to the test to be replicated numerically.

In the stent COMP, the minimum geometrical unit and the functional unit representative of the tensile test coincide, representing 1/32 of the whole stent, as shown in Fig. 2b. In particular, to match the whole stent behavior, the displacement applied on the unit had to be multiplied by 4 while the resulting force by 8. On the other hand, in the stent ABS, the minimum functional unit for the tensile test represents 1/6 of the whole stent in the circumferential direction (Fig. 2d). Although a smaller geometrical unit can be recognized, no other functional reductions can be applied due to the design-specific deformation fashion in this test, which is greatly dependent on the longitudinal coordinate. In this case, to match the whole stent behavior, the displacement applied on the unit coincided with that of the stent while the resulting force had to be scaled by a factor of 6. No mismatch is present in this case since the constraint applied to the whole stent is the same as the unit (Fig. 3a).

To mimic a uniaxial tensile test on the whole stent, both functional units were constrained as reported in Table 1.

**Table 1:** Boundary conditions for the uniaxial tensile tests on the functional units. See Fig. 2 for a clear picture of the surfaces. All boundary conditions refer to a cylindrical coordinate system, whose origin coincides with the stent centerline: *r*, *θ*, and *z* are the radial, circumferential and longitudinal directions, respectively

Surfaces	Boundary conditions
A1, A2	Uθ=0, ROTr=0, ROTz=0
Z1	Uz=Uθ=Ur=0, ROTr=0, ROTθ=0
Z2	Uz=ΔL, ROTr=0, ROTθ=0

### Ni–Ti Constitutive Parameters

The constitutive model available in Abaqus 2019/Standard (Dassault Systemes, SIMULIA Corp., RI) for super-elastic materials was used in this study.^6^ In addition to the Poisson’s ratio (*ν*), which is usually set as 0.3 according to the literature,^8^ the FE solver requires eight parameters to describe the material non-linear behavior at a certain temperature, namely the Young’s moduli of the austenite and martensite (*E*_A_ and *E*_M_ (MPa)), the amplitude of the transformation plateaus (*ε*_L_ (−)), the four stress values, defining the start and finish of the forward (*A*→*S*) and the reverse (*S*→*A*) transformations (*σ*_SAS_, *σ*_FAS_, *σ*_SSA_, *σ*_FSA_ (MPa)) in tension and the start stress for the forward transformation in compression (*σ*^c^_SAS_, (MPa), due to the material asymmetry).

For the identification process, it was decided to work on parameters that non necessarily correspond to the Abaqus inputs parameters. In particular, a parameter *H* (MPa) was defined as the slope of the transformation plateau during loading, defined as:

$$H=\frac{{\sigma }_{FAS}-{\sigma }_{SAS}}{\frac{{\sigma }_{FAS}}{{E}_{M}}+{\varepsilon }_{L}-\frac{{\sigma }_{SAS}}{{E}_{A}}}$$

Moreover, a parameter *α* (−), representing an index of the tension/compression asymmetry, was defined as:

$$\alpha =\frac{{\sigma }_{SAS}^{C}-{\sigma }_{SAS}}{{\sigma }_{SAS}^{C}+{\sigma }_{SAS}}.$$

### Identification Process on Virtual Cases

For each step, the process started by defining the lower and the upper bounds for all the admissible parameters. This range was based on literature analysis of possible values for medical-grade and super-elastic Ni–Ti (Table 2).^1,3,11,22,26,29^ For sake of simplicity, all the parameter ranges were normalized in the interval 0–1.

**Table 2:** Range of admissible values for each parameter: the lower bound was associated to 0 whilst the upper bound to 1.

*E*_A_ (MPa)	*σ*_SAS_ (MPa)	*H* (MPa)	*α* (−)	*E*_M_ (MPa)	σ_SSA_ (MPa)	σ_FSA_ (MPa)	*ε*_L_ (−)
40,000–60,000	200–700	50–2500	0–0.33	15,000–*E*_A_	80–300	50–270	0.04–0.065

For each phase, the corresponding influential parameters were sampled in the parametric space through the quasi-random SOBOL sequences.^33,35^ As a general rule, it was decided to consider $n$ points in each phase, where $n=20\times {N}_{p}$, and *N*_*p*_ is the number of design parameters. Following this rule, the initial sample space was 20 points for Phase I, 60 points for Phase II, and 80 points for Phase III.

Each point defined a unique set of material parameters and was numerically analyzed in a numerical simulation, described in “Stents FE models and functional units”. A loss function (*L*_2_) was calculated for each phase as an index of the discrepancy between the target behavior and the numerical case having material properties corresponding to the sampled point. *L*_2_ was defined as:

$$L2=\sqrt{{\sum }_{i=0}^{k}{\left({F}_{i}^{\mathrm{target}}-{F}_{i}^{\mathrm{point}}\right)}^{2}}$$

where $k$ is the number of simulation increments in the corresponding phase (i.e. 10, 40, or 50 depending on the phase), ${F}_{i}^{\mathrm{target}}$ is the target force value (e.g. the experimental output), and ${F}_{i}^{\mathrm{point}}$ is the force value resulting from the simulation, both evaluated at the $i$th time increment of the experiment or simulation. For each sample point in the parametric space, *L*_2_ will be different.

The loss function, calculated at each sample point, was used to construct a Gaussian Process (GP) surrogate model^17,24,30^ for each of the three phases. The GP model was used to identify the set of parameters minimizing the surrogate of the loss function for the considered phase, through a Limited-memory Broyden-Fletcher-Goldfarb-Shanno (L-BFGS) algorithm, a quasi-Newtonian optimization algorithm.^19^ When the solution of a phase was reached, subsequent phases used the parameters already identified in previous phases.

Two methods were used for assessing the validity of the surrogate models built for each phase. First, cross-validation, also referred to as the leave-one-out method, was performed.^16^ The method consists of excluding one of the samples for the construction of the GP model and comparing the GP prediction with the true response. This procedure is performed for all the samples. The built GP model is good if the plot showing the relationship between the actual values and the predictions identifies a linear behavior with a 45° slope. The second method for verifying the reliability of the GP models is the evaluation of the Standardized Cross-Validated Residuals (SCVR) obtained from the leave-one-out process.^16^ The SCVR related to each sample quantifies the number of standard errors by which the predicted value differs from the actual one. The GP model is considered valid if the SCVR values are generally small and contained in the interval [− 3,+ 3].

After the assessment of the validity of the GP models, a total-order sensitivity analysis on parameters^32,34^ was performed for Phase II and Phase III to evaluate the influence of each input parameter on the surrogate function for L2. The total-order sensitivity indices are related to the amount of variance of the output that is related to the parameter taken both singularly and in association with the other parameters.

The method was applied to two virtual tensile tests to assess its validity and effectiveness in a well-controlled scenario. In these cases, the COMP functional unit was associated with two literature-inspired sets of material properties, reported in Table 3. In particular, Mat-1 and Mat-2 refer to the work of Refs. 40 and 39 respectively.

**Table 3:** Literature-inspired sets of material properties used for the virtual cases^39,40^ and the normalized value referring to each parameter range.

	*E*_A_ (MPa)	*σ*_SAS_ (MPa)	*H* (MPa)	*α*	*E*_M_ (MPa)	*σ*_SSA_ (MPa)	*σ*_FSA_ (MPa)	*ε*_L_
Mat-1^40^	60,000	346	331.5	0.19	60,000	83	57	0.057
Mat-2^39^	45,000	310	430.7	0.19	15,000	100	75	0.0426

A numerical simulation of the COMP functional unit was performed with both material properties, for obtaining the target curves for the identification (Fig. 3b). The choice of employing the functional unit instead of the whole stent for the target curve was done to reduce the almost negligible, but existent, mismatch related to the use of the unit (Fig. 3a, black curves).

For all the twenty simulations involved in Phase I, it was not necessary to simulate the whole loading-unloading history, allowing an important reduction in the computational time. All the simulations consisted of the first 10 increments of the simulation (0.1 s of the total time of 1 s, where the results were sampled every 0.01 s).

An ad-hoc Matlab script (MathWorks, Inc., Natick, MA) allowed the automatic creation of an Abaqus input file per each of the sampled points in the parameters space (in the case of Phase I only *E*_A_). At the end of the computation, another script permitted the save of the force value at every increment of each simulated case, and then performed the calculation of the *L*_2_ associated with each sampled point.

Phase II consisted of time increments between the 11th and the 50th, requiring the computation of half the tensile test (loading phase only). The parameter identified in Phase I was set constant in all the simulations. Three parameters had to be sampled through the SOBOL sequence, namely *σ*_SAS_, *H*, and *α*. The same procedure of Phase I for the input file creation and, then, *L*_2_ calculation was applied.

Phase III was defined as the unloading phase, meaning from the 51st increment to the end of the simulation (100th). It was not possible to reduce the simulation time as in the previous cases. The parameters to sampled were *E*_M_, *σ*_SSA_, *σ*_FSA_, and *ε*_L_. A relation was enforced in the sampling process, as *σ*_SSA_ > *σ*_FSA_.

At the end of the post-processing of Phase III, the final set of identified parameters was evaluated in comparison with the known one, either Mat-1 or Mat-2.

### Uniaxial Tensile Tests on Real Devices

The average data available from the uniaxial tensile tests executed in Ref. 1 were used as the target of the identification process. Stent COMP and ABS underwent 4.5 mm and 6 mm, respectively, tensile tests in displacement control at 0.03 mm/s. It is interesting to remark that both the designs used in these experiments were laser-cut from the same source tube and underwent the same heat treatments, allowing to assume the same constitutive material for both the stents.

The tensile test was replicated numerically: both the stents were constrained at the extremities through two Multi-Point-Constraints (MPCs), where one was the control node for the applied displacement, while the other was constrained in all degrees of freedom.

The same process used in the virtual case was applied here. The experimental force-time curve was down-sampled to match a hundred increments of the numerical cases.

### Crush Tests and Verification

A crush test was performed using three samples for each stent design. The experiment was performed in a temperature-controlled water chamber (37.0 ± 0.1 °C) mounted on an MTS testing machine (Synergie 200H, MTS System Inc., Minneapolis, MN). The test was performed at 0.05 mm/s up to a negative displacement of 4 mm, then returned to zero.

The results in terms of average force-displacement curves were compared to the outputs of the simulations, performed on the previously described stent models with the same displacement boundary conditions applied to two 10 mm x 10 mm rigid plates (each of which discretized with 10×10 rigid 4-nodes elements, R3D4), to provide proof of the reliability of the identified parameters.

## Results

### Identification Process on Virtual Cases

**Figure 4:**
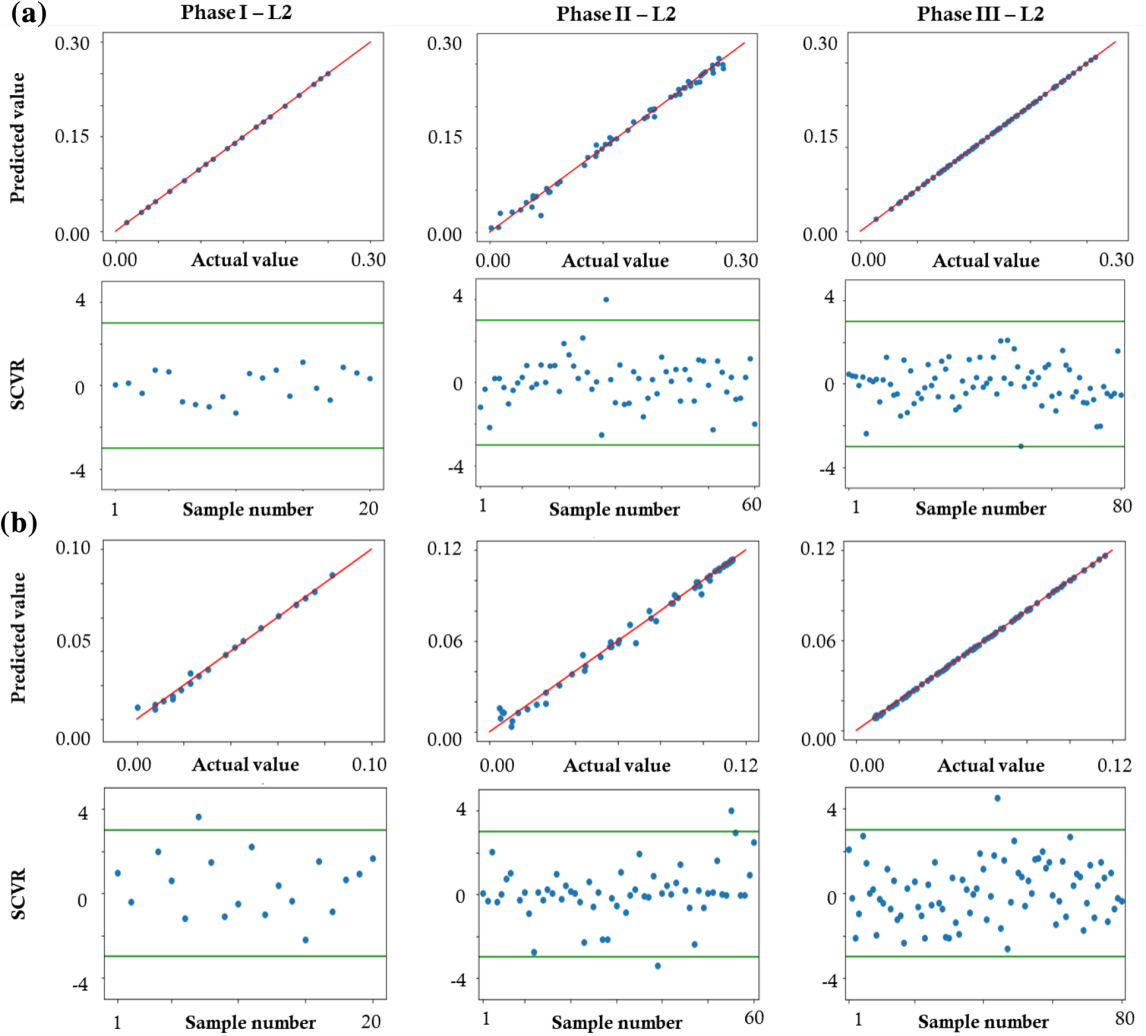
Assessment of the validity of the GP models for the identification of the three phases through the leave-one-out method and SCVR: (**a**) Mat-1 and (**b**) Mat-2.

The results of the process on the virtual cases were satisfactory. Figure 4a shows the leave-one-out and SCVR plots for the three identification phases for the first virtual case (Mat-1). In the leave-one-out, each plot represents the predicted and actual *L*_2_ value for each combination of the input parameters chosen in the design of the experiment. The same conclusion was drawn for Mat-2, here reported in Fig. 4b.

Tables 4 and 5 indicate the relative influence of the input parameters for Mat-1 for Phase II and Phase III, respectively, obtained with a total-order sensitivity analysis.^32,34^

**Table 4:** Ranking (total effects) of the input parameters of Phase II for Mat-1.

	*σ*_SAS_	*H*	*α*
Ranking	90.33 %	1.73 %	7.94 %

**Table 5:** Ranking (total effects) of the input parameters of Phase III for Mat-1.

	*E*_M_	*σ*_SSA_	*σ*_FSA_	*ε*_L_
Ranking	0.17 %	46.93 %	52.89 %	0.01 %

**Figure 5:**
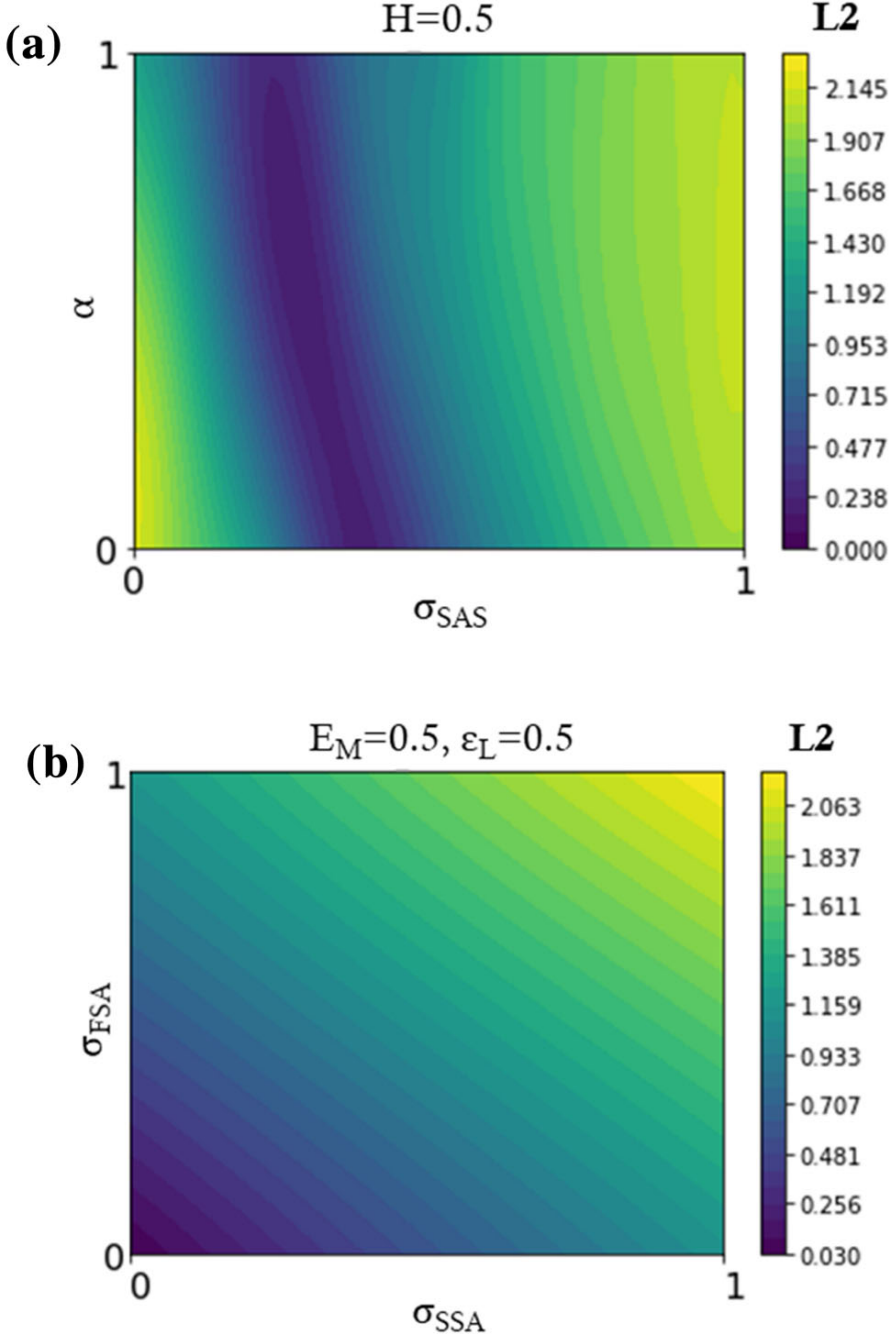
Representation of a slice of the response surface for Mat-1, showed according to the most relevant parameters, in the case of (**a**) Phase II (*H* fixed at 0.5) and (**b**) Phase III (*E*_M_ and *ε*_L_ fixed at 0.5).

Figure 5a represents a slice of the response surface created by the surrogate model for Phase II for Mat-1, showing the values of *L*_2_ as a function of the most relevant parameters (*σ*_SAS_ and *α*), with the parameter *H* fixed at the middle of its range. Figure 5b shows a slice of the response surface for Phase III for Mat-1, showing the values of L2 as a function of *σ*_FSA_ and *σ*_SSA_ with *E*_M_ and *ε*_L_ fixed in the middle of their ranges.

**Figure 6:**
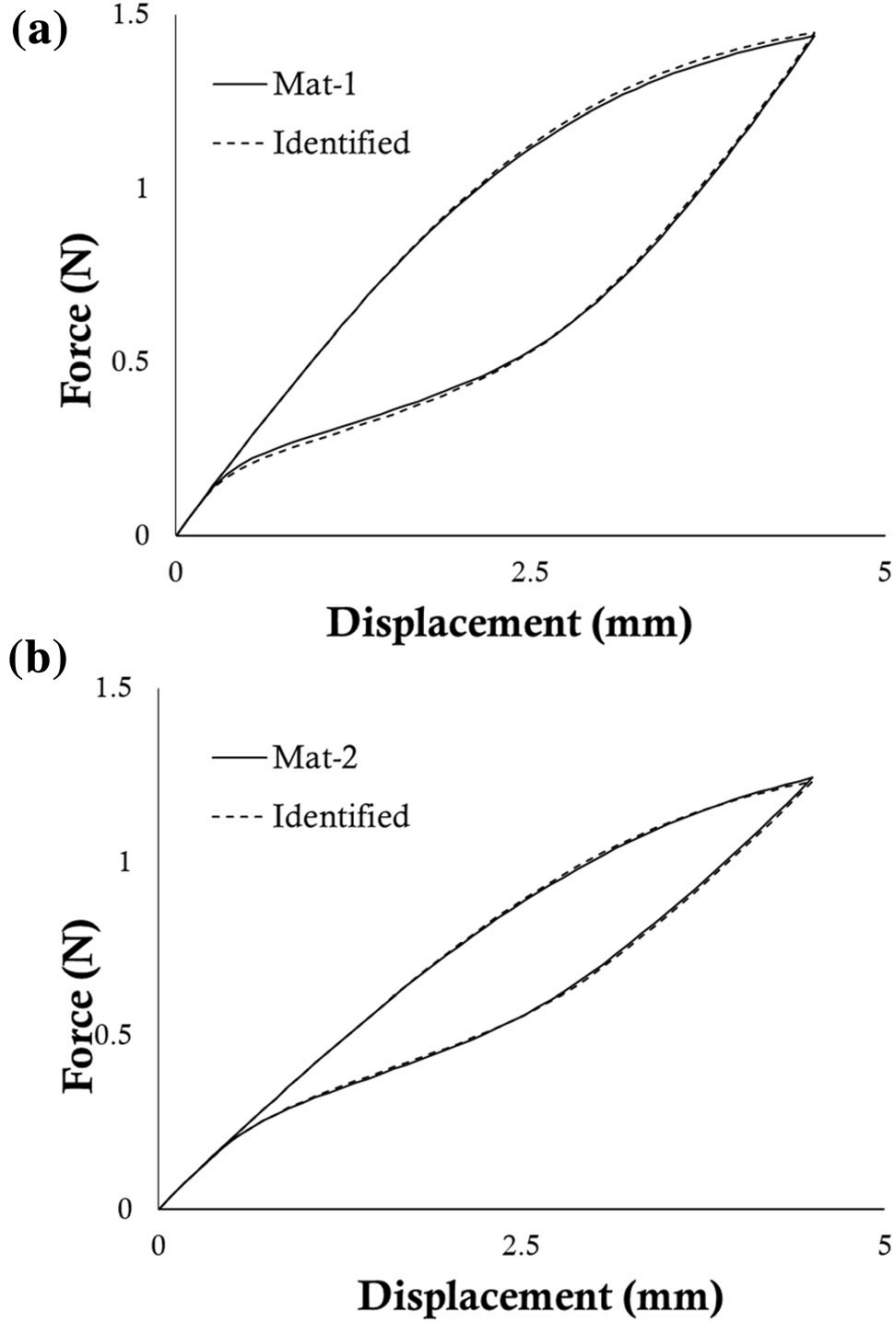
Comparison between the tensile force-displacement curves obtained using the target and identified parameters in case of (**a**) Mat-1 and (**b**) Mat-2.

The two sets of parameters identified in both the virtual cases are reported in Table 6. The numerical simulations of a tensile test on the unit performed using the identified sets are compared to the results obtained from the use of the actual Mat-1 and Mat-2 sets in Fig. 6. The computational time for each simulation of Phase I, II, and III was 15, 115, and 249 seconds respectively.

**Table 6:** Results of the identification process on virtual cases.

	*E*_A_ (MPa)	*σ*_SAS_ (MPa)	*H* (MPa)	*α*	*E*_M_ (MPa)	*σ*_SSA_ (MPa)	*σ*_FSA_ (MPa)	*ε*_L_
Mat-1	60,000	346.0	331.5	0.19	60,000	83.0	57.0	0.057
Identified	60,000	357.89	50	0.17	60,000	80	50	0.061
Mat-2	45,000	310.0	430.7	0.19	15,000	100	75	0.0426
Identified	45,000	331.6	436.8	0.09	15,000	126.3	73.2	0.04

### Uniaxial Tensile Tests on Real Devices

**Figure 7:**
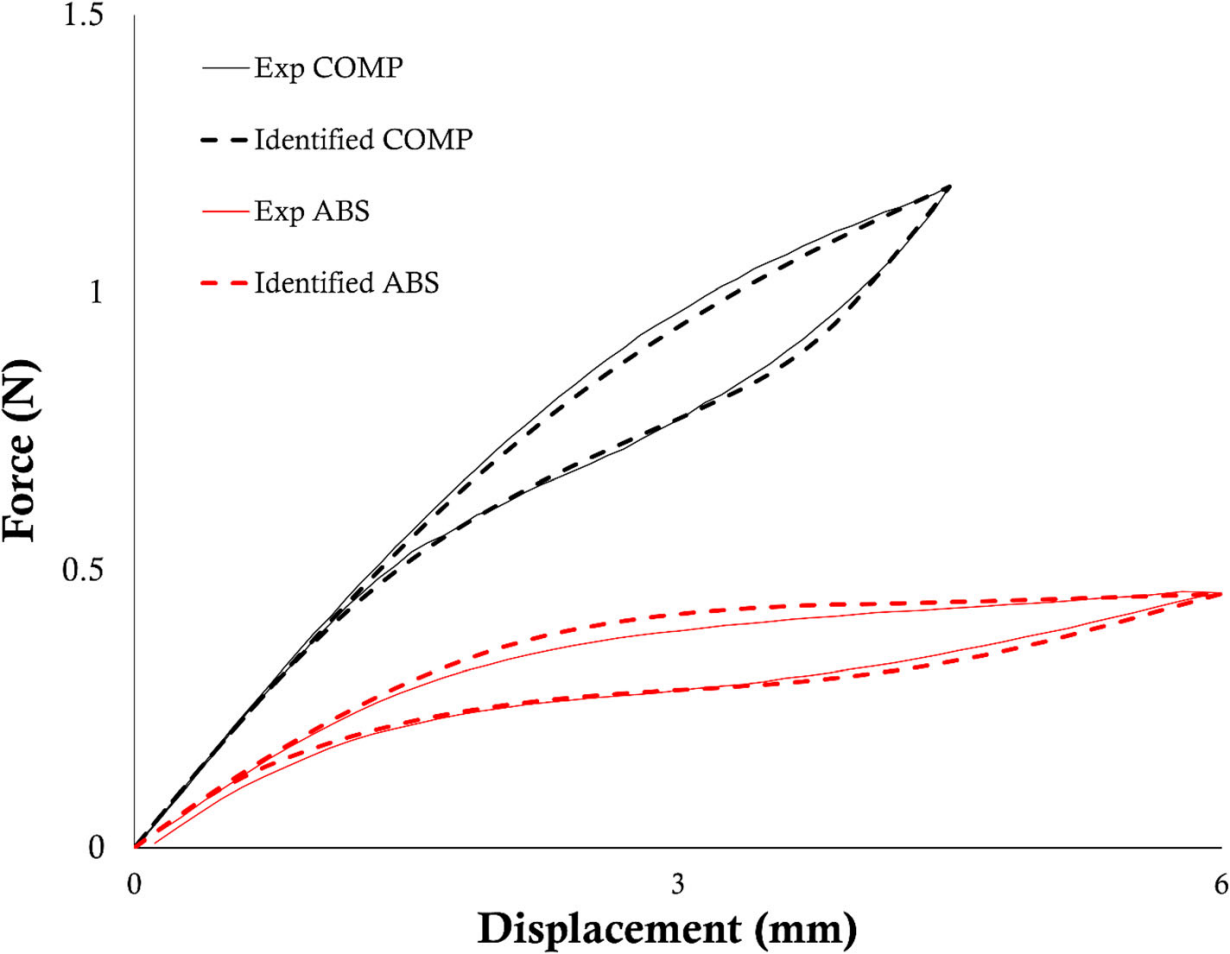
Comparison between the experimental (solid lines) tensile force-displacement curves and the numerical outputs (dashed lines) obtained using the identified set of parameters in case of stent COMP (black) and ABS (red).

The results of the identification process on the experimental tests involving the COMP and ABS stents are shown in Table 7. In particular, the identified sets were compared to the mechanical properties previously assessed on wire specimens made of the same material as the stents.^1^ The numerical simulations performed using these sets, compared with the experimental data, are visualized in Fig. 7. For the COMP design, the simulation time remained the same as in the virtual cases; as for the ABS design, the computational time for each simulation of Phase I, II, and III was 82, 644, and 2967 seconds respectively. This difference between the two models was due to the greater amount of elements composing the ABS unit.

**Table 7:** Results of the identification process on the real devices compared to the tensile properties assessed in previous work^1^ on wire specimens.

	*E*_A_ (MPa)	*σ*_SAS_ (MPa)	*H* (MPa)	*α*	*E*_M_ (MPa)	*σ*_SSA_ (MPa)	*σ*_FSA_ (MPa)	*ε*_L_
Wire specimens^1^	47,000	260	1625.22	–	22,000	140	80	0.045
Identified COMP	43,434	252.63	1984.21	0.33	15,000	195.79	154.21	0.04
Identified ABS	43,232	278.95	1081.58	0.26	43,232	195.79	50	0.04

### Crush Tests and Verification

**Figure 8:**
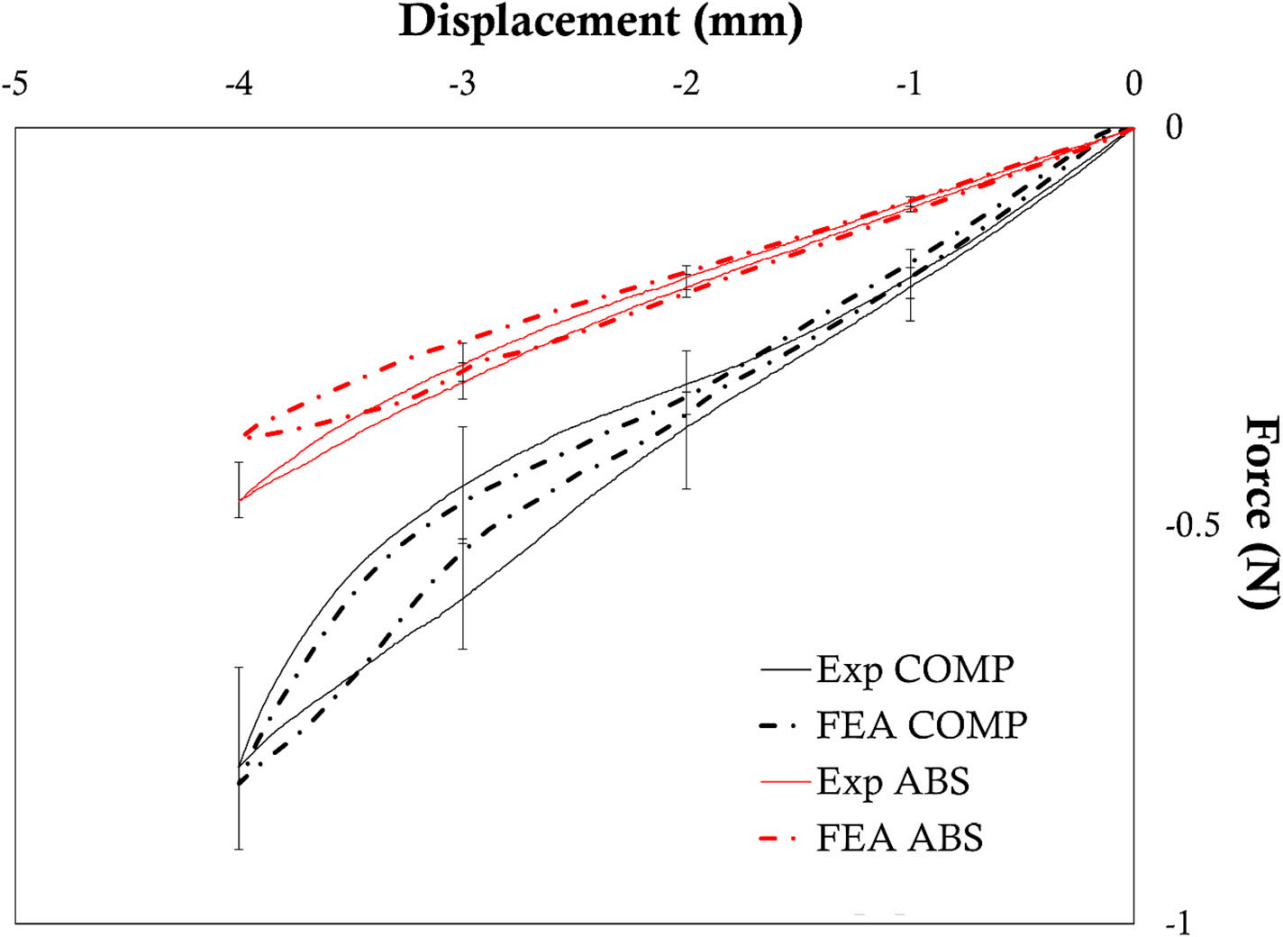
Comparison between the experimental (solid lines) curves from the crush test and the numerical outputs (dashed lines) obtained using the identified set of parameters in case of stent COMP (black) and ABS (red).

The comparison between the experimental force-displacement curves and the output of the simulations using the identified sets of parameters (Table 7) is shown in Fig. 8. Error bars, indicating the maximum and minimum value among the three tests, represent the experimental variability affecting the tests, which was higher in the case of the COMP design. Both the numerical curves correctly described the initial response, with a better match in the case of the COMP stent; in the case of the ABS design, the elastic response of the stent was well captured, with lower accuracy in correspondence of the maximum applied displacement.

## Discussion

The methodology here presented aims to propose a coupled experimental-numerical strategy that allows the realization of reliable Ni–Ti stent models. Such a method involves the identification of all the Ni–Ti material parameters from non-destructive experimental tests on a few stent samples. This could represent a valuable tool when no information is available on the mechanical properties of the constituent material (that is the case of clinicians or research institutes that desire to perform an investigation independently from the manufacturer).

Simple experiments involving the whole device, such as tensile tests, were proven suitable for achieving satisfactory data for the parameters identification; moreover, due to the Ni–Ti super-elasticity, these tests could be performed in a non-destructive fashion, to preserve and maximize the number of samples to test.

The manual tuning of the parameters, which is an effective first-tentative solution in absence of any data when dealing with standard elastic-plastic metals, in the Ni–Ti case, involving many constitutive parameters, can result in a laborious and ineffective process. Moreover, the complex stent design prevents any analytical consideration that could be performed in much simpler devices (such as spinal rods or nails for bone fixation), mandatorily requiring the FE tool for identification.

However, the particular Ni–Ti constitutive behavior allows for a step-wise process that cannot be exploited in the case of other materials.^2^

A surrogate modeling approach has been proposed as a new solution in this field and it was proven as a valuable solution to be coupled with non-destructive experiments for material identification.

The choice of dividing the whole process into three sub-phases was effective in reducing the dimensionality when (i) constructing the surrogate models, and (ii) searching/minimizing the surrogates. This leads to efficiencies in the estimation process and better estimates when compared to a single-phase identification of all eight parameters. This process also minimizes unrealistic parameter combinations (as only the relevant parameters are varied in individual phases) during sampling for surrogate construction, which minimizes failed (or unrealistically long) computational runs with odd combinations of the parameters. Finally, the employment of sub-phases reduces the computational time (-32% and -42% in the case of COMP and ABS, respectively) as simulations assessing the initial shorter sub-phases need not be run for the full range of displacement.

The two virtual cases were used as proof of the validity of the GP method, showing satisfactory results in the leave-one-out and SCVR tests (Fig. 4). Small discrepancies were found between the identified sets and the reference values, even if the visual comparison of the force-displacement curves in the reference and identified cases shows an almost perfect match (Fig. 6). This can be motivated by the different weights exhibited by the parameters, as shown from the ranking in Tables 4 and 5, in the definition of the overall response. Indeed, it is acknowledgeable that the role of the Ea is dominant, defining the first elastic response of the tested device. The results of the total-order sensitivity analysis on the parameters governing Phase II (Table 4) show how during loading the change in slope due to transformation (*σ*_SAS_) plays a major role in influencing the global response (90.33 %). A lower influence is related to the degree of tension-compression asymmetry (*α*, 7.94 %), while an almost negligible effect is associated with the *H* parameter (1.73 %). For this reason, once fixed the values of σ_SAS_ and α, a significant variation in the *H* parameter does not produce a relevant effect on the overall response. Therefore, the total-order sensitivity analysis explains why the visual comparison of the force-displacement curves in the reference and identified cases shows an almost perfect match (Fig. 6), even if the single value is different.

The results of the identification of the experimental cases showed good agreement between the experimental curve and the identified one. Moreover, the identified sets exhibited similarities in all the parameters values: it is worth pointing out how the COMP and ABS specimens were laser-cut from the same source tube of the wire specimens of the previous work.^1^ In this sense, it was expected to find two sets that could be similar to each other, and similar to the set of the wire specimens. Previous work^1^ highlighted the impossibility of assessing *σ*^C^_SAS_ from a pure tensile test on such thin specimens; moreover, poor literature is available on the assessment of Ni–Ti compressive properties on thin structures due to the intrinsic test difficulty due to instability. The results here presented showed an upgrade in that sense since the methodology could assess a value for *σ*^C^_SAS_ directly from the stent tensile test: this was feasible since the device’s deformation fashion is dominated by the bending of each v-strut, which contains information on both the tensile and compressive properties. However, it is credible to assume that the identified value of *σ*^C^_SAS_ compensates for the simplified numerical description of the compressive response in the Abaqus material module. Indeed, the tensile behavior is scaled based on the difference between σ_SAS_ and *σ*^C^_SAS_ to obtain the compressive curve: however, this does not allow to finely control the curve as in tension. The greatest effect for this compensation can be appreciated in the difference between the parameters describing the unloading phase of the tensile loading curve in COMP and ABS cases (namely Phase III, see *E*_M_, *σ*_SSA_, *σ*_FSA_ in Table 7) compared to the values from the wire specimens.^1^

It is expected that the use of a more refined constitutive model for the compressive behavior of the material could help to overcome this limitation: however, more parameters should be introduced for a more accurate description of the compressive response and, hence, their classification into the sub-phases revised.

However, the use of the crush tests to assess the global reliability of the identified sets of parameters in a different testing scenario, which involves a different local deformation fashion of the v-struts, proved the overall credibility of the virtual model (Fig. 8).

## Abbreviations

*E*_A_: The elastic modulus for the austenite phase
*E*_M_: The elastic modulus for the martensite phase
*ν*: Poisson’s ratio
*σ*_SAS_: Starting stress value for the forward phase transformation
*σ*_FAS_: Final stress value for the forward phase transformation
*σ*_SSA_: Starting stress value for the reverse phase transformation
*σ*_FSA_: Final stress value for the reverse phase transformation
*σ*^C^_SAS_: Starting stress value for the forward phase transformation in compression
*ε*_L_: The amplitude of the transformation plateaus
*α*: Index of tension/compression asymmetry
*H*: Slope of the transformation plateau

## References

[1] Allegretti D, Berti F, Migliavacca F, Pennati G, Petrini L (2018). Fatigue assessment of nickel-titanium peripheral stents: comparison of multi-axial fatigue models. Shape Mem. Superelasticity.

[2] Antonini L, Berti F, Isella B, Hossain D, Mandelli L, Pennati G, Petrini L (2021). From the real device to the digital twin: a coupled experimental-numerical strategy to investigate a novel bioresorbable vascular scaffold. PLoS ONE.

[3] Arrigoni M, Auricchio F, Cacciafesta V, Petrini L, Pietrabissa R (2001). Mechanical characterisation of orthodontic superelastic Ni-Ti wires. Strain.

[4] ASME (2018). V&V 40: Assessing Credibility of Computational Modeling Through Verification and Validation: Application to Medical Devices.

[5] Auricchio F, Constantinescu A, Conti M, Scalet G (2016). Fatigue of metallic stents: from clinical evidence to computational analysis. Ann. Biomed. Eng..

[6] Auricchio F, Taylor RL (1997). Shape-memory alloys: modelling and numerical simulations of the finite-strain superelastic behavior. Comput. Methods Appl. Mech. Eng..

[7] Azaouzi M, Makradi A, Belouettar S (2012). Deployment of a self-expanding stent inside an artery: a finite element analysis. Mater. Des..

[8] Berti F, Wang PJ, Spagnoli A, Pennati G, Migliavacca F, Edelman ER, Petrini L (2021). Nickel-titanium peripheral stents: Which is the best criterion for the multi-axial fatigue strength assessment?. J. Mech. Behav. Biomed. Mater..

[9] De Beule M, Mortier P, Carlier SG, Verhegghe B, Van Impe R, Verdonck P (2008). Realistic finite element-based stent design: the impact of balloon folding. J. Biomech..

[10] Bucsek AN, Paranjape HM, Stebner AP (2016). Myths and truths of nitinol mechanics: elasticity and tension-compression asymmetry. Shape Mem. Superelasticity.

[11] Conti M, Auricchio F, De Beule M, Verhegghe B (2009). Numerical simulation of Nitinol p eripheral stents: from laser-cutting to deployment in a patient specific anatomy. ESOMAT.

[12] Dordoni E, Meoli A, Wu W, Dubini G, Migliavacca F, Pennati G, Petrini L (2014). Fatigue behaviour of Nitinol peripheral stents: the role of plaque shape studied with computational structural analyses. Med. Eng. Phys..

[13] Drexel MJ, Selvaduray GS, Pelton AR (2006). The effects of cold work and heat treatment on the properties of nitinol wire. Proc. Int. Conf. Shape Mem. Superelastic Technol..

[14] Forrester AIJ, Keane AJ (2009). Recent advances in surrogate-based optimization. Prog. Aerosp. Sci..

[15] Harvey SM (2011). Nitinol stent fatigue in a peripheral human artery subjected to pulsatile and articulation loading. J. Mater. Eng. Perform..

[16] Jones DR, Schonlau M, Welch WJ (1998). Efficient global optimization of expensive black-box functions. J. Glob. Optim..

[17] Keane AJ, Nair PB (2005). Computational Approaches for Aerospace Design: The Pursuit of Excellence.

[18] Lagoudas D, Hartl D, Chemisky Y, Machado L, Popov P (2012). Constitutive model for the numerical analysis of phase transformation in polycrystalline shape memory alloys. Int. J. Plast..

[19] Liu DC, Nocedal J (1989). On the limited memory BFGS method for large scale optimization. Math. Program. Ser. B.

[20] MacTaggart JN, Phillips NY, Lomneth CS, Pipinos II, Bowen R, Timothy Baxter B, Johanning J, Matthew Longo G, Desyatova AS, Moulton MJ, Dzenis YA, Kamenskiy AV (2014). Three-dimensional bending, torsion and axial compression of the femoropopliteal artery during limb flexion. J. Biomech..

[21] Maleckis K, Deegan P, Poulson W, Sievers C, Desyatova A, MacTaggart J, Kamenskiy A (2017). Comparison of femoropopliteal artery stents under axial and radial compression, axial tension, bending, and torsion deformations. J. Mech. Behav. Biomed. Mater..

[22] Mckelvey AL, Ritchie RO (1999). Fatigue-crack propagation in nitinol: a shape-memory and superelastic endovascular stent material fatigue-crack propagation in Nitinol, a shape-memory and superelastic endovascular stent material. J. Biomed. Mater. Res..

[23] Morrison TM, Hariharan P, Funkhouser CM, Afshari P, Goodin M, Horner M (2019). Assessing computational model credibility using a risk-based framework: application to hemolysis in centrifugal blood pumps. ASAIO J..

[24] Pant S, Bressloff NW, Limbert G (2012). Geometry parameterization and multidisciplinary constrained optimization of coronary stents. Biomech. Model. Mechanobiol..

[25] Pelton AR (2011). Nitinol fatigue: a review of microstructures and mechanisms. J. Mater. Eng. Perform..

[26] Pelton AR, Fino-decker J, Vien L, Bonsignore C, Saffari P (2013). Rotary-bending fatigue characteristics of medical-grade Nitinol wire. J. Mech. Behav. Biomed. Mater..

[27] Petrini L, Bertini A (2020). A three-dimensional phenomenological model describing cyclic behavior of shape memory alloys. Int. J. Plast..

[28] Petrini L, Trotta A, Dordoni E, Migliavacca F, Dubini G, Lawford PV, Gosai JN, Ryan DM, Testi D, Pennati G (2016). A computational approach for the prediction of fatigue behaviour in peripheral stents: application to a clinical case. Ann. Biomed. Eng..

[29] Rebelo N, Fu R, Lawrenchuk M (2009). Study of a nitinol stent deployed into anatomically accurate artery geometry and subjected to realistic service loading. J. Mater. Eng. Perform..

[30] Sacks J, Welch WJ, Mitchell TJ, Wynn HP (1989). Design and analysis of computer experiments. Stat. Sci..

[31] Saigal A, Fonte M (2011). Solid, shape recovered “bulk” Nitinol: Part I-Tension-compression asymmetry. Mater. Sci. Eng. A.

[32] Saltelli A (2002). Making best use of model evaluations to compute sensitivity indices. Comput. Phys. Commun..

[33] Santner TJ, Williams BJ, Notz WI (2003). The Design and Analysis of Computer Experiments.

[34] Sobol IM (1993). Sensitivity estimates for nonlinear mathematical models. Math. Model. Comput. Exp..

[35] Sobol IM, Kucherenko S (2005). Global sensitivity indices for nonlinear mathematical models and their Monte Carlo estimates. Math. Comput. Simul..

[36] Stoeckel D, Pelton A, Duerig T (2004). Self-expanding nitinol stents: Material and design considerations. Eur. Radiol..

[37] Viceconti M, Juarez M, Curreli C, Pennisi M, Russo G, Pappalardo F (2019). POSITION PAPER: credibility of in silico trial technologies—a theoretical framing. IEEE J. Biomed. Heal. Informatics.

[38] Viceconti M, Pappalardo F, Rodriguez B, Horner M, Bischoff J, Musuamba Tshinanu F (2020). In silico trials: verification, validation and uncertainty quantification of predictive models used in the regulatory evaluation of biomedical products. Methods.

[39] Wu W, Pott D, Mazza B, Sironi T, Dordoni E, Chiastra C, Petrini L, Pennati G, Dubini G, Steinseifer U, Sonntag S, Kuetting M, Migliavacca F (2016). Fluid—structure interaction model of a percutaneous aortic valve: comparison with an in vitro test and feasibility study in a patient- specific case. Ann Biomed Eng.

[40] Wu W, Qi M, Liu XP, Yang DZ, Wang WQ (2007). Delivery and release of nitinol stent in carotid artery and their interactions: a finite element analysis. J. Biomech..

